# DesHDAP2 Shows Significant Synergy with Conventional Antibiotics, Despite Its Relatively Low Potency

**DOI:** 10.1021/acsomega.5c04806

**Published:** 2025-12-08

**Authors:** Anastasija Vasilijevic, Josefina Reyes Fernández, Brianna Perry, Clare Gibson, Louise E. O. Darling, Donald E. Elmore

**Affiliations:** † Biochemistry Program, 8456 Wellesley College, 106 Central St., Wellesley, Massachusetts 02481, United States; ‡ Department of Biological Sciences, 8456 Wellesley College, 106 Central St., Wellesley, Massachusetts 02481, United States; § Department of Chemistry, 8456 Wellesley College, 106 Central St., Wellesley, Massachusetts 02481, United States

## Abstract

Antimicrobial peptides (AMPs) represent a potential tool in combating increasing antibiotic resistance in bacteria. One promising application of AMPs is their use in antibacterial cocktails along with conventional, small-molecule antibiotics. Here, we investigate the potential antibacterial synergy of our novel histone-derived AMP, DesHDAP2. Consistent with previous studies, DesHDAP2 has relatively low antimicrobial activity on its own, particularly against Gram-negative bacteria. However, DesHDAP2 demonstrates antibacterial synergy with four antibiotics representing several different mechanisms of action, kanamycin, tetracycline, levofloxacin, and polymyxin B. Combinations of DesHDAP2 with these antibiotics also did not show any appreciable cytotoxicity against eukaryotic cells. These results highlight the promise of DesHDAP2 in combination therapy. Moreover, they emphasize the potential application of AMPs that have relatively low activity on their own in therapeutic cocktails with other antibiotics.

## Graphical abstract

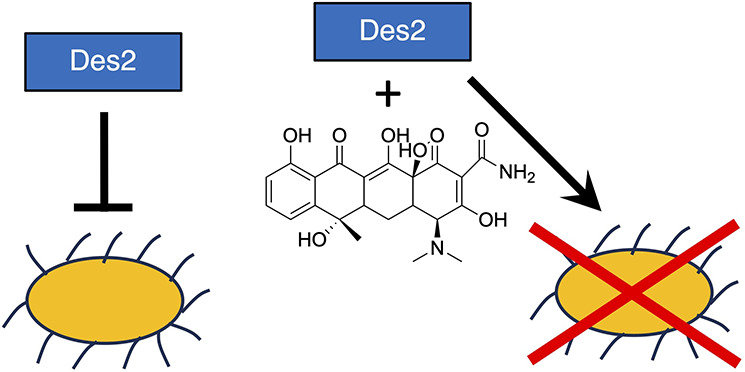

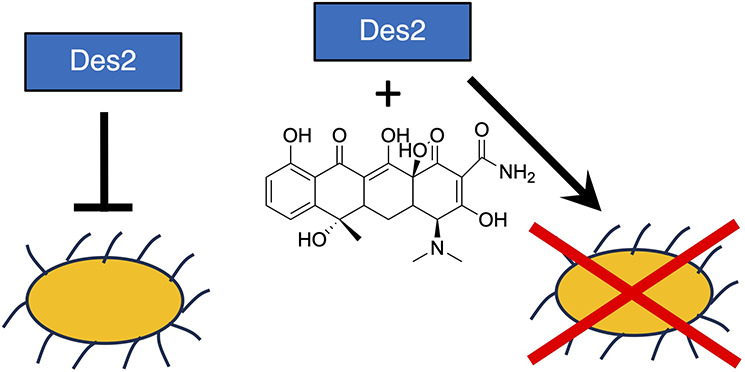

## Introduction

Antibiotic-resistant bacteria have become an increasingly acute health concern. The World Health Organization (WHO) reported in 2019 that antimicrobial resistance was the cause of 700,000 deaths and estimated that this number will rise to 20 million by 2050.^1^ In the United States, antimicrobial resistance currently costs over 55 billion dollars annually.^2^ Even though antimicrobial resistance has become a worldwide problem, posing a threat to lives and the economy, the development of new antibacterial agents has slowed down in recent decades.^3^ Thus, there is a pressing need to both develop new therapeutics and determine novel ways to employ already existing antibiotics.

Antimicrobial peptides (AMPs) represent one potential strategy to address the need for new antibiotic therapies. AMPs are relatively short amino acid sequences (∼10–50 amino acids) that have antimicrobial properties. In the past decades, these peptides have been isolated from various organisms, including vertebrate and invertebrate animals, plants, bacteria, and fungi, and they are already part of the natural defense of most living organisms. AMPs have proven to be active against a wide range of microorganisms, including Gram-positive and Gram-negative bacteria, protozoa, yeast, fungi, and viruses.^4^ In addition to being potential therapies on their own, studies have highlighted the potential synergistic effects in drug “cocktails” made by mixing AMPs with conventional, small-molecule antibiotics.^5–8^ For example, many well-characterized peptides, such as magainin 2,^9^ tacheplesin-III,^10^ and LL-37,^11^ have shown significant synergy with common antibiotics, such as those in the tetracycline and carbapenem families. It has also been hypothesized that combination therapies may be less prone to inducing antibacterial resistance since bacteria would need to simultaneously develop or acquire resistance to more than one active agent.^12,13^

In previous work, we designed three novel histone-derived antimicrobial peptides (HDAPs), DesHDAP1–3.^14^ As with other HDAPs, these three peptides do not all have the same mechanism of action. Like buforin II (BF2),^15–17^ DesHDAP1 is able to translocate into cells without causing significant membrane damage, ultimately causing cell death by its interaction with intracellular nucleic acids.^18^ Conversely, DesHDAP2 and DesHDAP3 appear to damage bacterial membranes,^14,18^ similar to hipposin and parasin.^19,20^ Despite their differing mechanisms, DesHDAP1 and DesHDAP3 showed appreciable antimicrobial activity and were, therefore, characterized and tested in subsequent research.^18,21–24^ However, DesHDAP2 showed significantly lower antibacterial activity and has not been the subject of any further study to date.^14,18^

In this work, we considered the potential for DesHDAP2 to be a synergistic agent in antibiotic/AMP cocktail mixtures, despite its relatively low baseline activity. To that end, we confirmed the low activity of DesHDAP2 against a series of Gram-positive and Gram-negative bacteria. We also found that a strategy of truncation, which has enhanced the activity for some histone-derived peptides, was not effective at increasing the activity of DesHDAP2. Nonetheless, despite its modest antibiotic activity, DesHDAP2 was effective as a synergistic agent with four different antibiotics that have differing mechanisms of action. Furthermore, we show that these DesHDAP2 and antibiotic mixtures do not exhibit enhanced cytotoxicity against human cells. In addition to highlighting interesting characteristics of DesHDAP2, these results emphasize the broad potential value of including AMPs with less promising activity in antibiotic cocktails.

## Results and Discussion

### Full-Length DesHDAP2 Has Low Antimicrobial Activity

We tested full-length DesHDAP2 for antimicrobial activity against Gram-positive (*Bacillus subtilis*, *Staphylococcus epidermidis*, and *Enterococcus raffinosus*), and Gram-negative (Top10 *Escherichia coli*, *Enterobacter aerogenes*, and *Serratia marcescens*) bacteria using radial diffusion assays (Figure 1). In these experiments, DesHDAP2 showed minimal activity against all Gram-negative strains. However, DesDHAP2 did show significant activity against the Gram-positive bacteria, although this potency was relatively low compared to that of other HDAPs considered in past work, such as BF2, parasin, hipposin, DesHDAP1, and DesHDAP3.^14–16,18,19,23,25^ Similarly, as discussed below, DesHDAP2 showed no activity against *B. subtilis* or *E. coli* in microbroth dilution assays up to a concentration of 125 μg/mL.

**Figure 1:**
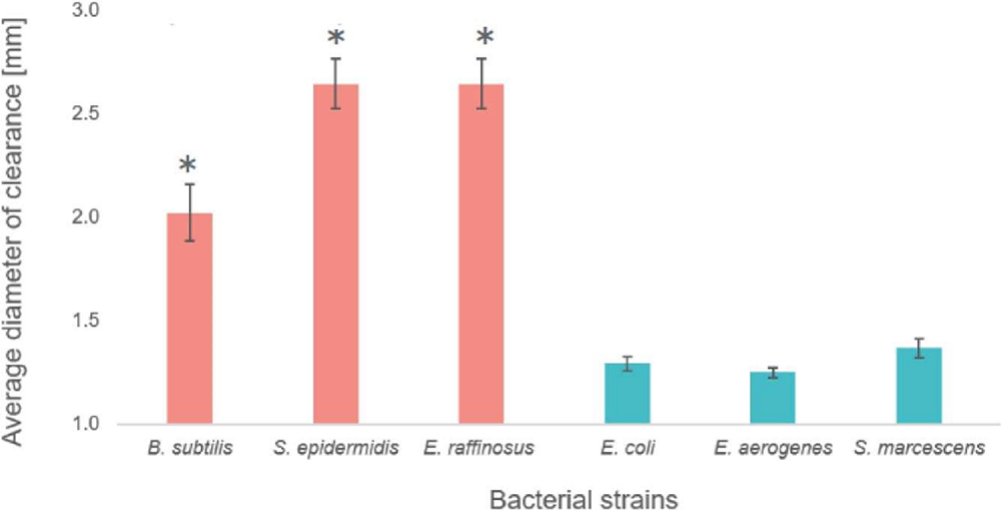
Results of radial diffusion assays for DesHDAP2 against various Gram-positive (salmon) and Gram-negative (teal) bacteria. Diameters are averaged over three technical replicates each from at least three independent biological experiments. Error bars represent the standard error. The concentration of DesHDAP2 was 250 μg/mL in all samples. Significance difference (*p* < 0.001) between clearance for a peptide and a well containing water (1 mm) is shown as *.

### Truncation Did Not Enhance DesHDAP2 Activity, and Only Minimal Truncations Retain Activity of the Full-Length Peptide

Building on our work with the full-length DesHDAP2 peptide, we subsequently considered the activity of DesHDAP2 variants truncated from the N- or C-terminus. These studies were performed for two reasons. First, previous work had demonstrated that truncations to buforin and DesHDAP1 actually could enhance the activity of some HDAPs.^26,27^ Second, even if they did not enhance activity, measuring the activity of truncations would provide insight into the minimum length of DesHDAP2 necessary for antibacterial activity (Table 1). Since cationic amino acids are often critical for AMP function,^28,29^ truncations were successively trimmed to an arginine or lysine residue. We used radial diffusion assays to determine the activity of each truncated peptide against the panel of Gram-positive and Gram-negative bacteria considered for the full-length peptide.

**Table 1:** Amino Acid Sequences of Full-Length DesHDAP2 Truncations^a^

peptide	amino acid sequence
DesHDAP2 full length	H**R**Y**R**PGTVAL**R**EI**RR**YQ**K**ST
DesHDAP2 1–18	H**R**Y**R**PGTVAL**R**EI**RR**YQ**K**
DesHDAP2 1–15	H**R**Y**R**PGTVAL**R**EI**RR**
DesHDAP2 1–14	H**R**Y**R**PGTVAL**R**EI**R**
DesHDAP2 1–11	H**R**Y**R**PGTVAL**R**
DesHDAP2 1–4	H**R**Y**R**
DesHDAP2 2–20	**R**Y**R**PGTVAL**R**EI**RR**YQ**K**ST
DesHDAP2 4–20	**R**PGTVAL**R**EI**RR**YQ**K**ST
DesHDAP2 11–20	**R**EI**RR**YQ**K**ST
DesHDAP2 14–20	**RR**YQ**K**ST
DesHDAP2 15–20	**R**YQ**K**ST

a Cationic residues are bolded.

Against Gram-negative strains, all truncations showed no measurable activity with diameters of clearance similar to those of the full-length peptide (Supplemental Figures 1–3). However, we were able to clearly determine the section of the peptide most critical for the observed activity against Gram-positive bacteria (Figure 2 and Supplemental Figures 4 and 5). In most cases, the truncations removing the fewest amino acids from either the C- or N-termini, 1–18 and 2–20, maintain antibacterial activity equivalent to that of full-length DesHDAP2. Similarly, both the 1–18 and 2–20 truncations did not show any activity up to 125 μg/mL in microbroth dilution assays with *E. coli* and *B. subtilis*, the same result as the full-length peptide. However, any larger truncations cause a significant drop in the measured diameter of the clearance. The precise effect of truncation on antibacterial activity did show some species dependence, as the cleavage of a single N-terminal amino acid disrupted activity in *E. raffinosus* (Supplemental Figure 5) but not *B. subtilis* (Figure 2) or *S. epidermis* (Supplemental Figure 4). Regardless, overall, the peptide was very sensitive to truncations, and no truncations significantly enhanced activity. Since a similar change in activity is observed when removing a single positive charge from either side of the peptide, we hypothesize that the decrease in activity for truncations is more likely due to a decrease in overall peptide charge than to a specific structural change. However, future work could consider the origin of increased sensitivity to N-terminal truncation against *E. raffinosus*. Since the truncations did not enhance activity and any potential cost savings from using a DesHDAP2 variant with the small allowed truncations would be minimal, we decided to use the full-length version of DesHDAP2 for subsequent synergy experiments.

**Figure 2:**
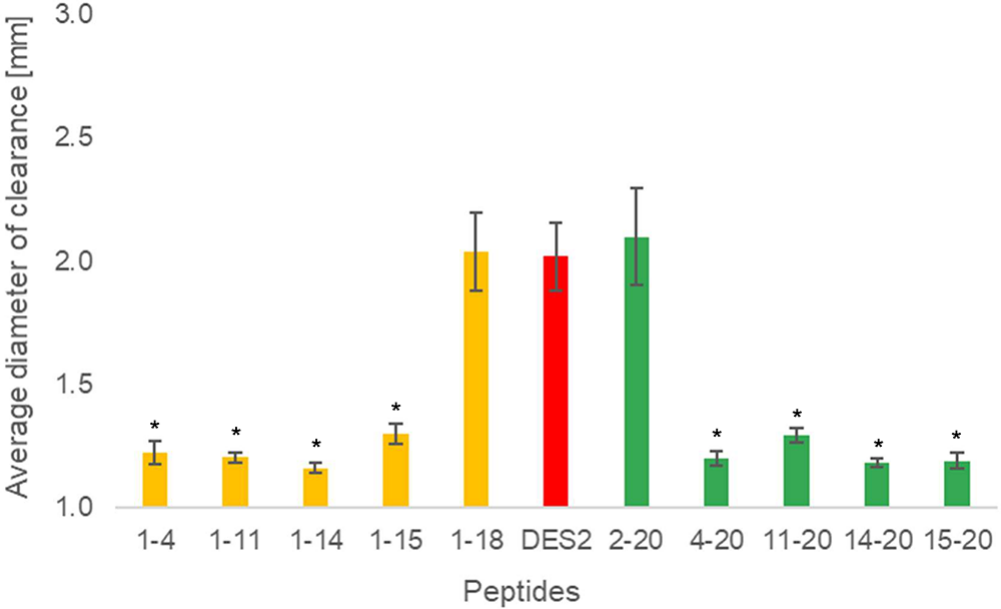
Results of radial diffusion assays for C- (yellow) and N-terminal (green) truncated DesHDAP2 against *B. subtilis*. Full-length DesHDAP2 is abbreviated in figure labels as Des2. Diameters are averaged over three technical replicates each from four independent biological experiments. Error bars represent standard error. The concentration of peptides was 250 μg/mL in all samples, and the size of a well was 1 mm. Significance difference (*p* < 0.001) between clearance for a truncated peptide and the full length peptide is shown as *.

### DesHDAP2 Shows Synergy with Conventional Antibiotics

Although DesHDAP2 showed only minimal antibacterial activity, we were curious whether it could be effective in a cocktail mixture with a conventional, small-molecule antibiotic. To this end, we decided to consider the effectiveness of DesHDAP2 combinations with kanamycin, levofloxacin, tetracycline, and polymyxin B against *B. subtilis* and *E. coli*. We chose to focus on these two strains for the initial studies for a few reasons. First, given the clear difference in activity of DesHDAP2 against Gram-positive and Gram-negative bacteria, we wanted to consider its potential synergy against one strain from each of these categories. We then selected *B. subtilis* and *E. coli* from each category due to their common use as model strains in much of the literature on HDAPs and other AMPs.^14,16,19,25^ These antibiotics were chosen to span a range of mechanisms, including translation inhibition (kanamycin and tetracycline), DNA replication inhibition (levofloxacin), and membrane disruption (polymyxin B).

As a first step to determine synergy, we measured a minimum inhibitory concentration (MIC) of DesHDAP2 and each antibiotic agent, independently, against *B. subtilis* and *E. coli* using a microbroth dilution assay (Table 2, Supplemental Figure 6). DesHDAP2 did not lead to bacterial clearance in this experiment at the highest concentration tested (125 μg/mL). Thus, a value of 125 μg/mL was used in the fractional inhibitory concentration index (FICI) calculation for the synergistic activity of DesHDAP2 with the conventional antibiotics. This approach was conservative in determining synergy, as it led to greater (i.e., less synergistic) FICI values than if we had used the actual (>125 μg/mL) MIC of DesHDAP2 in these calculations.

**Table 2:** Average MIC of DesHDAP2 and Conventional Antibiotics against *E. coli* and *B. subtilis* Measured in Microbroth Dilution Assays^a^

antimicrobial agent	average MIC against *B. subtilis* (μg/mL)	average MIC against *E. coli* (μg/mL)
DesHDAP2	>125	>125
kanamycin	3.125	50
levofloxacin	5	0.2
tetracycline	15.625	10
polymyxin B	2000	65

a Values are averaged over three technical replicates each from four independent biological experiments.

We investigated potential synergistic activity in checkerboard assays with *B. subtilis* and *E. coli*. In these assays, we measured whether combinations of DesHDAP2 and an antibiotic at varying concentrations inhibited bacterial growth. In these experiments, we considered twofold diluted concentrations of conventional antibiotics starting from their MIC and of DesHDAP2 starting from 25 μg/mL. Synergy was quantitatively determined by calculating the FICI value (eq 1–3)^30,31^ for the DesHDAP2+antibiotic combination with bacterial clearance showing the highest potential synergy (Supplemental Figure 7).

In standard interpretations of checkerboard experiments, combinations of drugs that exhibit FICI values less than 0.5 are considered to be synergistic.^30,31^ This appears to be a reasonable threshold as some peptide·antibiotic combinations that show effectiveness when used with in vivo systems may even have somewhat higher FICI values, such as combinations of the DJK-5 and 1018 peptides with ciprofloxacin used against a mouse abscess model.^6^ Using this definition, DesHDAP2 showed synergy with all four antibiotics against *B. subtilis* in our experiments (Figure 3). More strikingly, consistent synergy was observed against *E. coli* for kanamycin and levofloxacin and generally with polymyxin B, despite the very low level of activity of the peptide alone against that bacterium and other Gram-negative strains.

**Figure 3:**
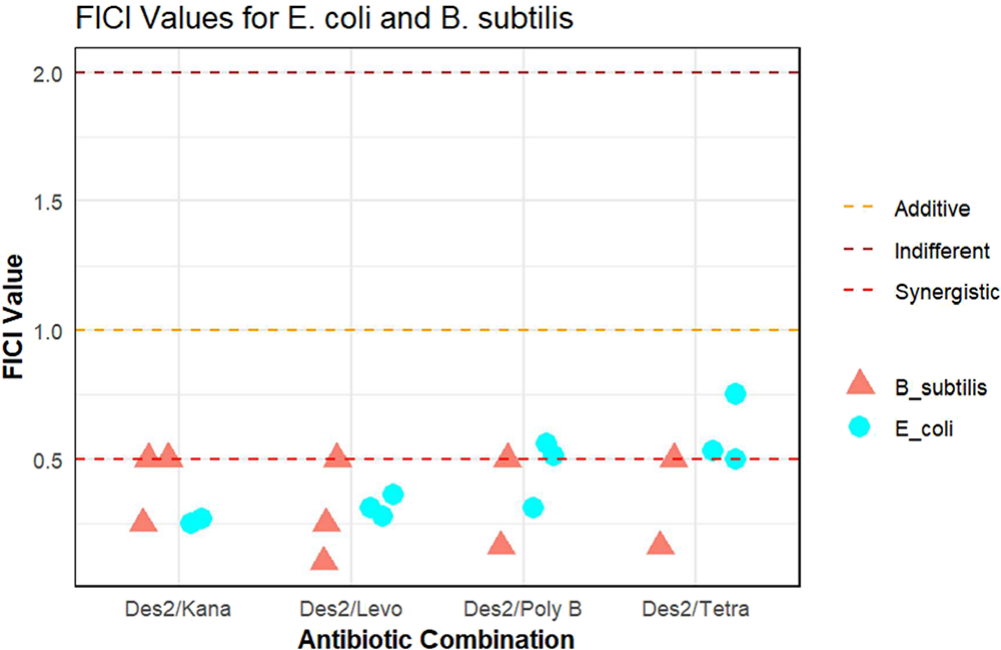
FICI for combinations of DesHDAP2 (abbreviated in figure labels as Des2) with different conventional antibiotics (abbreviated in figure labels as Kana, Levo, Poly B, and Tetra for kanamycin, levofloxacin, polymyxin B, and tetracycline, respectively) against *B. subtilis* and *E. coli* in checkerboard assays. FICI was determined at 24 h. Symbols show FICI values from independent biological experiments performed with *E. coli* (circles) or *B. subtilis* (triangles). Dashed lines represent the upper limit for FICI categories (synergistic, FICI ≤ 0.5; additive, 0.5 < FICI ≤ 1; and indifferent, 1 < FICI ≤ 2).

### Cocktails of DesHDAP2 with Conventional Antibiotics Do Not Show Eukaryotic Cytotoxicity

Since we observed promising synergy between DesHDAP2 and conventional antibiotics, we wanted to consider whether any of these combinations led to undesirable cytotoxicity against human cells. To this end, we measured the impact of DesHDAP2 mixtures with all four antibiotics considered on HEK 293 cells in MTS assays. Healthy cells with metabolic activity can metabolize the MTS reagent to form a product with absorption at 490 nm. In these experiments, we exposed cells to mixtures of DesHDAP2 and conventional antibiotics with concentrations greater than those needed to observe synergistic activity in checkerboard assays.

In these assays, the measured absorbance values of the DesHDAP2+antibiotic cocktails are similar to those of cells treated with water and drastically different from those of cells treated with a high concentration of DMSO (20%), which is toxic to cells (Figure 4). The absorbance values thus indicate that the tested DesHDAP2+antibiotic combinations are not cytotoxic and further support that cocktails are a promising therapeutic strategy.

**Figure 4:**
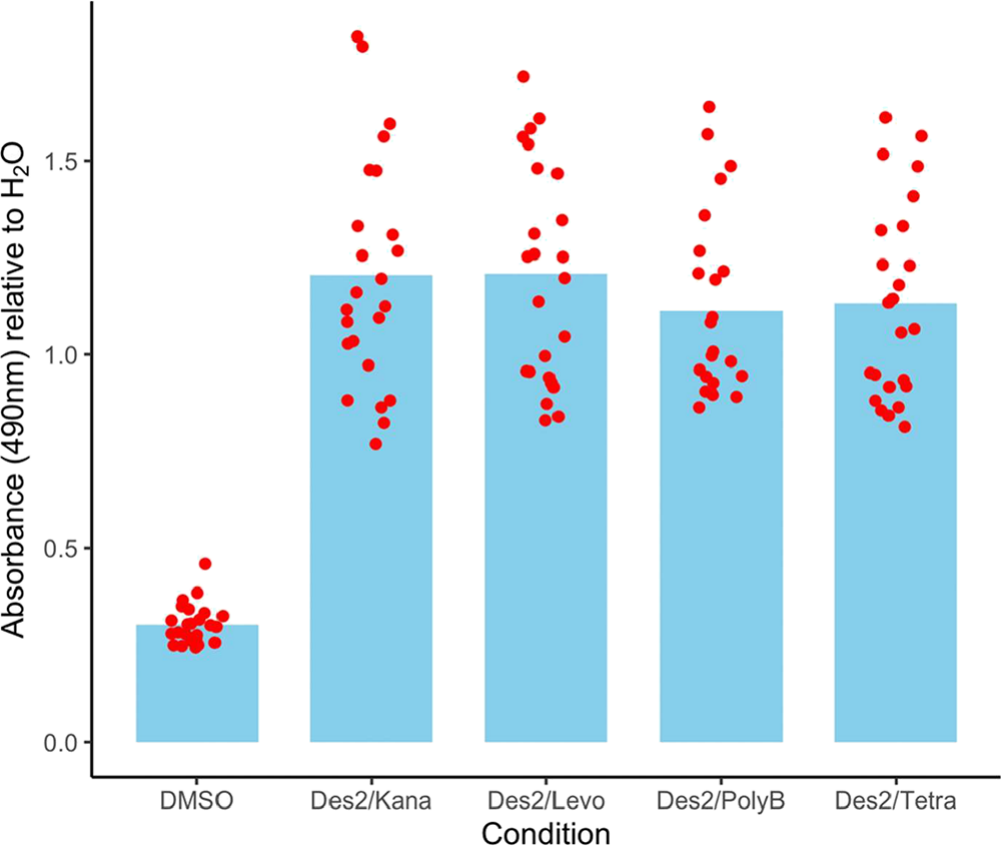
Absorbance at 490 nm for HEK 293 cells in MTS assays exposed to DMSO or cocktails of DesHDAP2 (abbreviated in figure labels as Des2) with a conventional antibiotic (abbreviated in figure labels as Kana, Levo, Poly B, and Tetra for kanamycin, levofloxacin, polymyxin B, and tetracycline, respectively). Values are expressed relative to the absorbance of cells exposed to water. Absorbances were measured for eight technical replicates each from three independent biological experiments. All measurements are shown as red dots with average values depicted by bars. Cocktails used a combination of 62.5 μg/mL DesHDAP2 with kanamycin (50 μg/mL), levofloxacin (10 μg/mL), polymyxin B (500 μg/mL), or levofloxacin (10 μg/mL). A DMSO concentration of 20% was used.

## Conclusion

Here, we have demonstrated that DesHDAP2 is able to work synergistically with a range of conventional antibiotics, despite its relatively low antibacterial activity when used alone. Moreover, synergistic DesHDAP2+antibiotic combinations do not appear to have appreciable cytotoxicity in human cells. These results support further investigation and engineering of DesHDAP2 to optimize its potential value as an adjuvant in antibiotic therapy. In particular, it would be interesting to investigate the mechanistic origins of their synergistic effects. Previous work implied that the antimicrobial activity of DesHDAP2 on its own was a result of its ability to disrupt bacterial membranes.^14^ Thus, we hypothesize that synergy might result from DesHDAP2 at low concentrations causing enough membrane damage to facilitate entry of other antibiotics even if that damage was not sufficient to inhibit bacterial growth on its own. It would be particularly interesting for future studies to consider expanding on this work to demonstrate whether these DesHDAP2/antibiotic synergies are observed with a broader range of strains such as others considered in this work or ESKAPE pathogens that often develop antibiotic resistance. Future work could also design experiments to more robustly explore whether better synergy is observed for DesHDAP2 with particular antibiotics that more closely share a mechanism of action—or even those that have more disparate mechanisms.

While the results presented in this paper have interesting implications about DesHDAP2, they also emphasize the potential value of antimicrobial peptides in cocktail therapy approaches even if those peptides may not be sufficiently potent for therapeutic use on their own. Given that many broad-spectrum AMPs have relatively high MIC values, this type of combination therapy may represent a particularly promising avenue for the ultimate clinical applications of AMPs. In fact, this approach could supplement attempts to enhance the activity of antibiotics with significant resistance in clinical settings.^32,33^ To that end, our results support researchers more broadly considering the potential synergies that could arise with other AMPs that otherwise have not been considered promising drug targets due to their relatively lower antimicrobial activity in isolation.

## Methods

### Peptide Design and Synthesis

Peptides were synthesized and purified to >95% by GenScript (Piscataway, NJ). Peptides were dissolved in deionized water and sterilized by passing through a 0.2 μM syringe filter. Concentrations were confirmed by taking tyrosine absorbance readings at 280 nm (ε=1490 M^–1^ cm^–1^) in triplicate using a ThermoScientific NanoDrop 2000 or Jasco UV/vis spectrophotometer. Peptide solutions were stored at −20 °C. Each experiment was conducted with a freshly thawed peptide solution, and peptide solutions were limited to approximately three freeze–thaw cycles.

### Antibiotics

All of the antibiotics used in this study (tetracycline, kanamycin, levofloxacin, and polymyxin B) were purchased from Sigma-Aldrich. Measured amounts of antibiotic were dissolved in sterile water and passed through a 0.2 μM syringe filter. Aliquots were stored at −20 °C and were limited to approximately three freeze–thaw cycles before disposal.

### Bacterial Species and Handling

The bacteria species and strains used in this study were: Top10 *E. coli* containing a pET plasmid with ampicillin resistance (Invitrogen Top10), *S. marcescens* (Carolina Biosciences #155554A), *E. aerogenes (*ATCC #51697*), E. raffinosus* (ATCC #49464), *S. epidermidis* (ATCC #14990) and *B. subtilis* (ATCC #6501). For radial diffusion, microbroth dilution, and checkerboard assays, bacterial cultures in tryptic soy broth (TSB) were started from individual colonies picked from agar plates and grown overnight (≈16–18 h) in a shaking incubator at 37 °C. Overnight bacterial cultures were diluted into fresh TSB and grown at 37 °C to an optical density (OD_600_) of 0.5–0.7. Bacteria were pelleted via centrifuge at 1500*g* at room temperature for 10 min, washed once with cold sterile sodium phosphate buffer (10 mM Na_3_PO_4_, 100 mM NaCl, pH 7.4), then pelleted and resuspended again in sodium phosphate buffer.

### Radial Diffusion Assay (RDA)

A radial diffusion assay^34^ was used to determine the antimicrobial activity of DesHDAP2 and its peptide truncations. A resuspended bacterial culture prepared as described above was added to melted TSB underlay agarose gel that was made using 10 mM Na_3_PO_4_, 1% TSB (BD DIFCO) v/v, and 1% agarose (VWR) w/v and adjusted to a final pH of 7.4. Agar was cooled in a water bath to a temperature of 45 °C. Sufficient culture was added to provide a final concentration of 4 × 10^6^ CFU/mL of bacteria. Then, 10 mL of underlay gel was poured into a Petri dish and allowed to solidify. 1 mm wells in the agar were made using Pasteur pipettes and a bleach trap. 2 μL of 250 μg/mL peptide was added to each well. The plate was incubated for 3 h at 37 °C, after which 10 mL of molten overlay gel (2.4% w/v TSB, 1% w/v agarose) was added to the plate and allowed to solidify. The plate was then incubated overnight (≈16–18 h) at 37 °C. The diameter of bacterial clearance was measured at 7× magnification using a magnifying eyepiece. Data for each bacterium and peptide combination were collected from plates from at least three different overnight cultures.

### Microbroth Dilution Assay

Antimicrobial activity was assessed using the microbroth dilution assay.^35^ A resuspended bacterial culture prepared as described above was mixed with deionized water to reach a final bacterial concentration of 4 × 10^6^ CFU/mL. A twofold dilution series of peptides in deionized water was prepared. 10 μL of the serial dilutions were added to 90 μL of the 4 × 10^6^ CFU/mL bacterial solution in a 96-well plate. DesHDAP2 peptide was added at 1250 μg/mL starting concentration, resulting in a 125 μg/mL highest working concentration. Maximum concentrations used varied for each antibiotic depending on their relative potency. After incubating for 1 h at 37 °C, 50 μL of TSB was added to each testing well. After incubation for 24 h at 37 °C, growth inhibition was determined visually to assess MIC.

### Checkerboard Assay

A checkerboard assay was used to determine the combined activity of two antimicrobial agents, DesHDAP2 and a conventional, small-molecule antibiotics.^30,31^ In a 96-well plate, bacteria were exposed to different combinations of a dilution series of both DesHDAP2 and an antibiotic (Supplemental Figure 7). A resuspended bacterial culture prepared as described above was mixed with deionized water to reach a final bacterial concentration of 4 × 10^6^ CFU/mL. Specifically, DesHDAP2 twofold dilutions were used across rows, and twofold dilutions of an antibiotic were used across columns. In each well, 10 μL of the peptide and 10 μL of the antibiotic dilution are mixed to provide the highest working concentrations of 250 μg/mL of DesHDAP2 and the MIC of the antibiotic. Control wells contained either 20 μL of sterile deionized water or 20 μL of 100% DMSO as a vehicle control. 80 μL of the 4 × 10^6^ CFU/mL bacterial solution then was added to all wells. The plate was incubated for an hour at 37 °C, after which 100 μL of 2× TSB (60% w/v) was added to all wells. After incubation for 24 h at 37 °C, growth inhibition were determined visually. At least two independent biological experiments were performed for each DesHDAP2+antibiotic combination.

Synergy was quantified by calculating the FICI for each plate. This was done using the following formulas:

$$\mathrm{FICI}={\mathrm{FIC}}_{\mathrm{DesHDAP2}}+{\mathrm{FIC}}_{\mathrm{antibiotic}}$$ 1

$${\mathrm{FIC}}_{\mathrm{DesHDAP2}}=\frac{\mathrm{MIC of DesHDAP2 in combination}}{\mathrm{MIC of DesHDAP2 alone}}$$ 2

$${\mathrm{FIC}}_{\mathrm{antibiotic}}=\frac{\mathrm{MIC of antibiotic in combination}}{\mathrm{MIC of antibiotic alone}}$$ 3

Based on the calculated FICI, the combined activity of the peptide+antibiotic mix can be categorized as synergistic, additive, indifferent, or antagonistic using the following categories: synergistic (FICI ≤ 0.5); additive (0.5 < FICI ≤ 1); indifferent (1 < FICI ≤ 2); and antagonistic (FICI ≥ 2).^30,31^ The well with the maximum synergy (lowest FICI) was reported for each plate.

### MTS Cytotoxicity Assay

HEK 293 cells (ATCC CRL-1573) were used for the MTS assay.^36^ HEK 293 cells were handled following BSL-2 protocols approved by the Wellesley College Institutional Biosafety Committee (IBC). In brief, cells were maintained in complete growth medium (EMEM, 10% fetal bovine serum, 1% penicillin–streptomycin) in a T-75 cell culture flask at 37 °C, 95% humidity, and 5% CO_2._ Cells were fed with fresh medium every 1–2 days and passaged when they reach 70–90% confluence, approximately every 5–7 days.

After incubation, media was aspirated and refreshed for each well, and 20 μL of the CellTiter 96 AQueous One Solution Reagent (Promega G3580) was added and allowed to incubate for 1–4 h (37 °C, 95% humidity, 5% CO_2_). Absorbance of each well was read at 490 nm on a Molecular Devices SpectraMax M3 microtiter plate reader.

For the MTS assay, 90 μL of culture containing 40,000 cells was plated in each well of a 96-well plate and incubated for 4–6 h. Following the incubation, a total of 20 μL of controls or peptide+antibiotic cocktails were added. These contained: 20 μL of sterile deionized water, 20 μL of 100% DMSO (for a working concentration of 20%), or 10 μL of DesHDAP2 and 10 μL of antibiotic. The final, effective concentration of DesHDAP2 was 62.5 μg/mL in each well, and final, effective concentrations of antibiotics were tetracycline (5 μg/mL), kanamycin (50 μg/mL), levofloxacin (10 μg/mL), and polymyxin B (500 μg/mL). Cells were incubated overnight (≈18 h), and then the wells were aspirated and replaced with fresh medium. Then, 20 μL of the CellTiter 96 AQueous One Solution Reagent (Promega G3580) was added. The plate was put back in the incubator, and after 4 h, the absorbance at 490 nm was measured with a Molecular Devices SpectraMax M3 microtiter plate reader.

## Supplementary Material



## Notes

Additional radial diffusion and microbial broth dilution assay data for full-length and truncated variants of DesHDAP2 and schematic of checkerboard assay (PDF)

## ⊥

University of Massachusetts Amherst, Physical Sciences Building, 690N Pleasant St, Amherst, Massachusetts01003,United States
