# Supplementary material for: DesHDAP2 Shows Significant Synergy with Conventional Antibiotics, Despite Its Relatively Low Potency
Source: ACS Omega. 2025 Dec 8;10(50):61224–30. doi: 10.1021/acsomega.5c04806 (PMC12750394; doi:10.1021/acsomega.5c04806)
Supplement: Supplementary file 1 [file ao5c04806_si_001.pdf]

Supporting Information for: DesHDAP2 Shows Significant Synergy with Conventional Antibiotics, Despite its Relatively Low Potency

Anastasija Vasilijevic[1], Josefina Reyes Fernández[1], Brianna Perry[1], Clare Gibson[1], Louise E. O. Darling*[1,2], Donald E. Elmore*[1,3]

[1] Wellesley College, Biochemistry Program, 106 Central St., Wellesley, MA 02481, USA

[2] Wellesley College, Department of Biological Sciences, 106 Central St., Wellesley, MA 02481, USA

[3] Wellesley College, Department of Chemistry, 106 Central St., Wellesley, MA 02481, USA

* Corresponding authors:
Louise E. O. Darling: ldarling@wellesley.edu
Donald E. Elmore: delmore@wellesley.edu

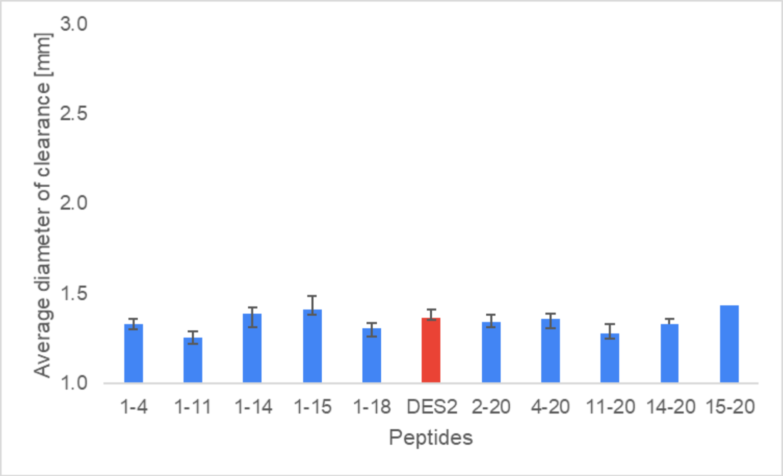

Figure S1. Results of radial diffusion assays for C- and N- terminus truncated DesHDAP2 against *S. marcescens*. The concentration of peptides was 250 µg/mL in all samples. Diameters are averaged over 3 technical replicates from three independent experiments. Error bars represent standard error. The size of a well is 1mm.

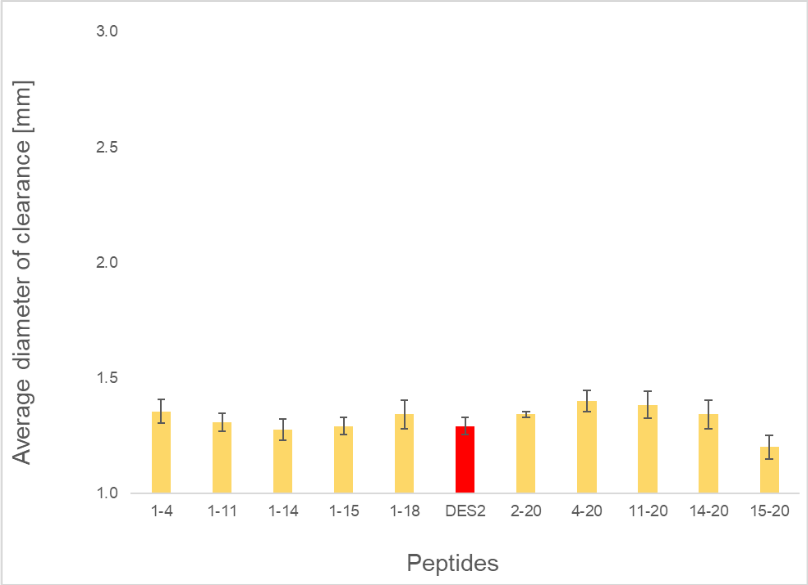

Figure S2. Results of radial diffusion assays for C- and N- terminus truncated DesHDAP2 against *E.coli*. The concentration of peptides was 250 µg/mL in all samples. Diameters are averaged over 3 technical replicates from three independent experiments. Error bars represent standard error. The size of a well is 1mm.

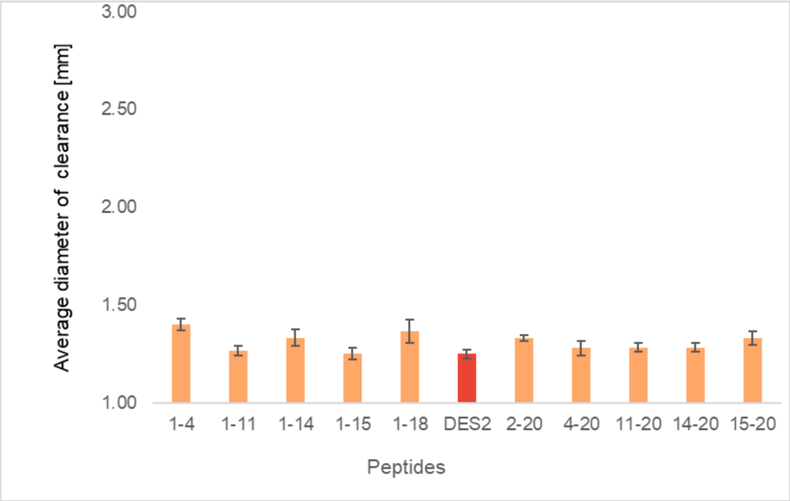

Figure S3. Results of radial diffusion assays for C- and N- terminus truncated DesHDAP2 against *E. aerogenes*. The concentration of peptides was 250 µg/mL in all samples. Diameters are averaged over 3 technical replicates from three independent experiments. Error bars represent standard error. The size of a well is 1mm.

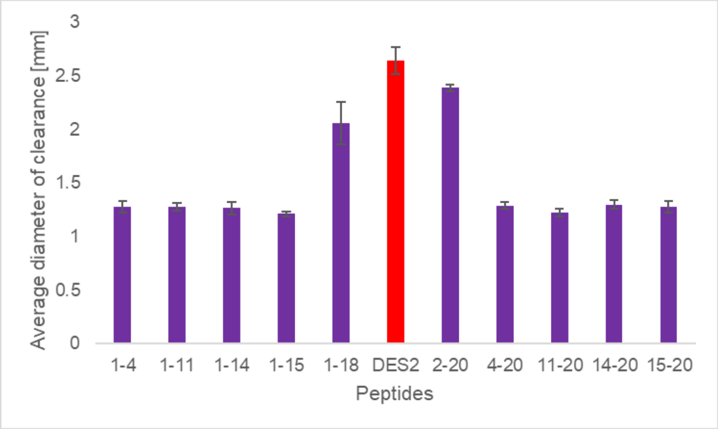

Figure S4. Results of radial diffusion assays for C- and N- terminus truncated DesHDAP2 against *S. epidermidis*. The concentration of peptides was 250 µg/mL in all samples. Diameters are averaged over 3 technical replicates from three independent experiments. Error bars represent standard error. The size of a well is 1mm.

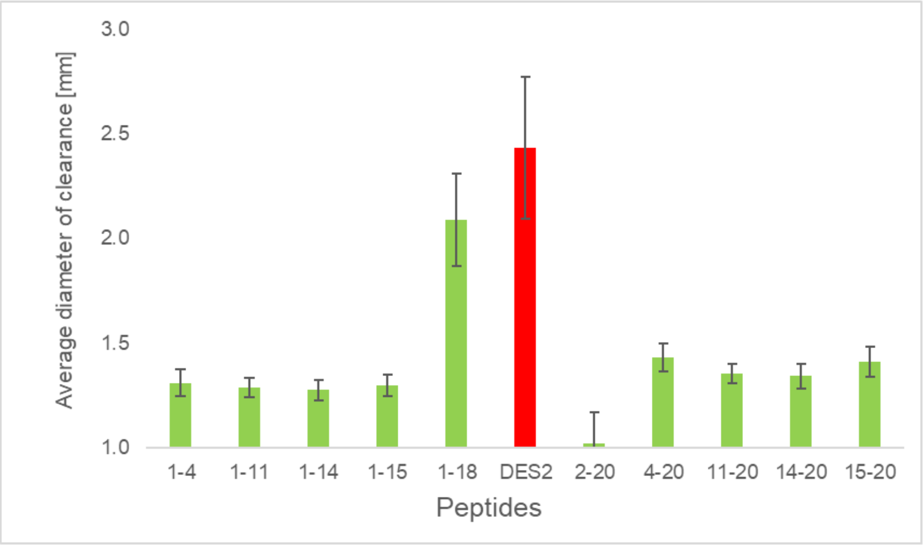

Figure S5. Results of radial diffusion assays for C- and N- terminus truncated DesHDAP2 against *E. raffinosus*. The concentration of peptides was 250 μg/mL in all samples. Diameters are averaged over 3 technical replicates from three independent experiments. Error bars represent standard error. The size of a well is 1mm.

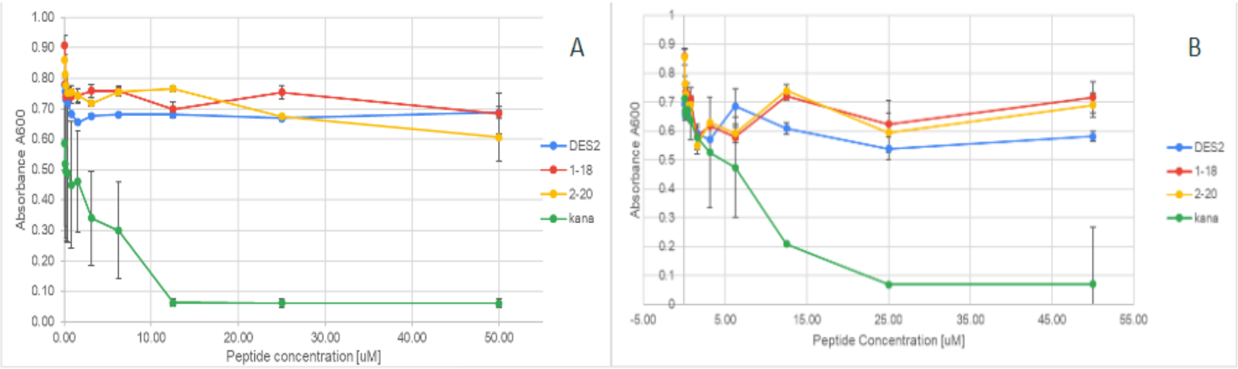

Figure S6. Microbroth dilution assay of full-length DesHDAP2, truncations 1-18 and 2-20, and Kanamycin (positive control) against *E.coli* (A) and *B. subtilis* (B). 50 μM corresponds to a concentration of 125 μg/mL. The average absorbance (OD600) was calculated for each peptide at each concentration along with the standard error of the mean. Absorbance values under 0.2 equate to bacterial clearance. The plot represents an average of n=3 biological replicates, each with n=3 technical replicates, for each peptide tested. Error bars represent standard error over all nine technical replicates.

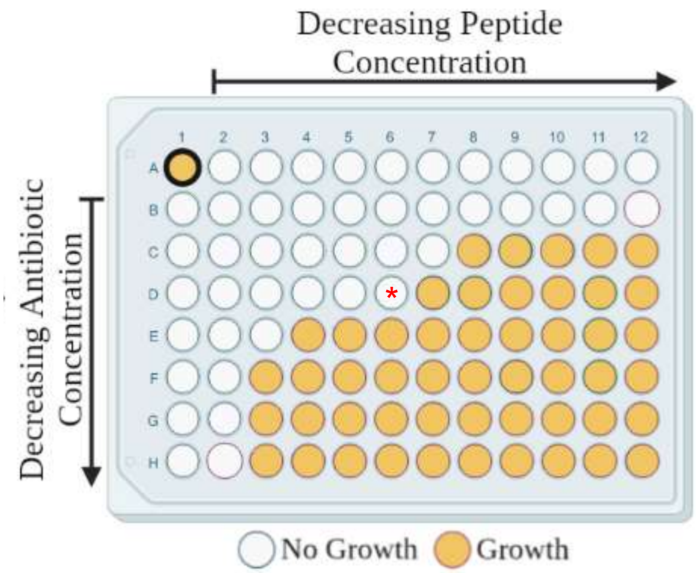

Figure S7. Schematic of checkerboard assay exposing bacteria to varying concentrations of peptide and antibiotic to determine potentially synergistic combinations. The combination demonstrating the greatest synergy that would be used to calculate FICI values is denoted by a red asterisk.
